# Need to Assess On‐Demand Versus Continuous Treatment in Allergic Rhinitis in ARIA‐EAACI Guidelines

**DOI:** 10.1111/all.70498

**Published:** 2026-08-27

**Authors:** Jean Bousquet, Bernardo Sousa‐Pinto, Maria J. Torres, Mario Morais‐Almeida, Ludger Klimek, Giorgio Ciprandi, Robert Naclerio, Torsten Zuberbier

**Affiliations:** ^1^ Charité – University Medical Center Berlin, Corporate Member of Freie Universität Berlin and Humboldt‐Universität zu Berlin, Institute of Allergology Berlin Germany; ^2^ Fraunhofer Institute for Translational Medicine and Pharmacology ITMP, Immunology and Allergology Berlin Germany; ^3^ MEDCIDS – Department of Community Medicine, Information and Health Decision Sciences, Faculty of Medicine University of Porto Porto Portugal; ^4^ CINTESIS@RISE – Centre for Health Technology and Services Research, Health Research Network, Faculty of Medicine University of Porto Porto Portugal; ^5^ Allergy Unit, Regional University Hospital of Málaga Málaga University, ARADyAL Málaga Spain; ^6^ Allergy Center CUF Descobertas Hospital Lisbon Portugal; ^7^ Department of Otolaryngology, Head and Neck Surgery Universitätsmedizin Mainz Mainz Germany; ^8^ Center for Rhinology and Allergology Wiesbaden Germany; ^9^ Department of Medicine and Health Sciences University of Molise Campobasso Italy; ^10^ Department of Otolaryngology – Head and Neck Surgery Johns Hopkins University Baltimore Maryland USA

To the Editor,

Most physicians treating patients with allergic rhinitis (AR) indicate that many patients use on‐demand treatment. However, the frequency of on‐demand versus continuous treatment use remains unclear.

Thus, one of the prioritised new questions of the Allergic Rhinitis and its Impact on Asthma‐European Academy of Allergy and Clinical Immunology (ARIA‐EAACI) guidelines 2026–2027 on AR is ‘Should continuous long‐term treatment vs. as‐needed treatment be used in patients with AR?’

We aimed to assess the relevance of this question. Although this has not been investigated in a general population study, there is some evidence in selected populations.

One first piece of evidence stems from MASK‐air studies. Although MASK‐air patients do not represent the general population, they can provide a glimpse into the patterns of medication use. Studies evaluating the behaviour of MASK‐air users suggest that patients frequently treat themselves as needed and according to their symptoms. Adherence to AR medication is likely to be poor, as investigated by two studies focussing on adherence.

In the first study, among 6949 unselected patients, 860 (12.4%) reported MASK‐air for 7 to 14 days. Adherence to treatment (Medication Possession Ratio > 70% and Proportion of Days Covered: 1–1.5) was observed in 5.2% of patients [1].

In the second study, among 11,713 European patients who reported at least one day of treatment with oral anti‐histamines (OAH), intranasal corticosteroids (INCS) or intranasal anti‐histamines + INCS (INAH + INCS), 1361 (11.6%) reported at least one full week of MASK‐air data in the pollen season (with medication adherence ranging from 31.7% to 38.5% depending on the treatment class). Only 484 (4.1%) reported at least one full month of MASK‐air data, with medication adherence ranging from 25.3% to 34.9% [2].

Adherence in these two studies was low. Importantly, these MASK‐air studies evaluated participants from several countries, and there were no major differences between countries. While this points to across‐country generalisability, it is important to emphasise that MASK‐air users are not representative of the general population, with a potential over‐representation of patients with higher access to healthcare and more concerned about their health. If this is the case, we may hypothesise that treatment adherence is even lower in the general population. However, this should be evaluated by studies performed in those populations, distinguishing between different chronicity (intermittent vs. persistent) and severity levels (mild vs. moderate–severe).

Another piece of evidence was obtained when assessing physicians that suffer from AR. During an EAACI meeting, adherence to AR treatment among allergists was assessed and found to be surprising: none of the 108 allergic physicians reported adherence [3]. After publishing this study, the first author (J.B.) informally repeated this procedure in over 200 conferences (unpublished data) and observed a very low adherence among allergists (less than 3%).

Those advocating for a continuous treatment point to the concepts of a priming effect of the nasal mucosa [4] and of minimal persisting inflammation [5, 6]. The priming effect suggests that treating patients before the start of the pollen season will improve symptoms during the pollen season in a mechanism associated with inflammation. Minimal persisting inflammation is closely dependent on allergen exposure and suggests that continuous treatment is needed since pollen exposure may vary during the season and inflammation may persist in days with low pollen counts. In addition, AR caused by perennial allergens is typically characterised by persistent inflammation. These concepts have been studied in nasal challenge and natural exposure models [4, 5].

However, while reducing nasal inflammation has been extensively tested in RCTs, a specific strategy of titrating treatment until a predefined state of ‘minimal inflammation’ is achieved has not been a focus in RCTs. In two RCTs, in a total of 40 children, cetirizine or fluticasone reduced minimal persistent inflammation.

Moreover, in AR, there is no universally accepted treatment target analogous to the ‘treat‐to‐target’ approach used in diseases like rheumatoid arthritis. We hypothesise that asthma multimorbidity, treatment satisfaction and adherence may be considered for an eventual ‘treat‐to‐target’ approach in rhinitis.

In conclusion, the available data, although obtained from selected populations (allergists and MASK‐air users), suggests that most patients with AR use their treatment on demand rather than continuously. Whether this real‐world treatment behaviour provides outcomes comparable to continuous treatment remains insufficiently studied and should be urgently addressed through dedicated guideline recommendations. These recommendations may vary according to (i) the presence of asthma, (ii) the type of medication, (iii) whether rhinitis is intermittent or persistent and (iv) whether it is mild or severe, depending on the results of the studies comparing these two approaches on outcomes such as symptom control, treatment satisfaction, use of co‐medication and impact of rhinitis in productivity and quality‐of‐life.

## Author Contributions

J.B. and B.S.‐P. have participated in the concept of the letter and in writing the original draft. M.J.T., M.M.‐A., L.K., G.C., R.N. and T.Z. have participated in the concept of the letter and in manuscript writing and revision.

## Funding

The authors have nothing to report.

## Conflicts of Interest

Dr. Bousquet reports personal fees from Cipla, Menarini, Mylan, Novartis, Purina, Sanofi‐Aventis, Teva, Noucor, other from KYomed‐Innov, other from Mask‐air‐SAS outside the submitted work. Dr. Naclerio reports personal fees from Lyra, personal fees from Sanofi, outside the submitted work. Dr. Zuberbier reports personal fees from Amgen, personal fees from AstraZeneca, personal fees from AbbVie, personal fees from ALK‐Abelló, personal fees from Almirall, personal fees from Astellas, personal fees from Bayer Health Care, personal fees from Bencard, personal fees from Berlin Chemie, personal fees from FAES Farma, personal fees from HAL Allergie GmbH, personal fees from Henkel, personal fees from Kryolan, personal fees from Leti, personal fees from L'Oreal, personal fees from Meda, personal fees from Menarini, personal fees from Merck Sharp & Dohme, personal fees from Novartis, personal fees from Nuocor, personal fees from Pfizer, personal fees from Sanofi, personal fees from Stallergenes, personal fees from Takeda, personal fees from Teva, personal fees from UCB, personal fees from Uriach, personal fees from Abivax, personal fees from Blueprint, personal fees from Celldex, personal fees from Celltrion, outside the submitted work; and Committee member, ‘Allergic Rhinitis and its Impact on Asthma’ (ARIA), Member of the Board, German Society for Allergy and Clinical Immunology (DGAKI), Head, European Centre for Allergy Research Foundation (ECARF), President, Global Allergy and Asthma Excellence Network (GA^2^LEN), and Member, Committee on Allergy Diagnosis and Molecular Allergology, World Allergy Organization (WAO). The other authors have nothing to disclose outside the submitted work.

## Data Availability

The data that support the findings of this study are available from the corresponding author upon reasonable request.

## References

[1] E. Menditto , E. Costa , L. Midao , et al., “Adherence to Treatment in Allergic Rhinitis Using Mobile Technology. The MASK Study,” Clinical and Experimental Allergy 49, no. 4 (2019): 442–460, 10.1111/cea.13333. 30597673

[2] B. Sousa‐Pinto , E. M. Costa , R. J. Vieira , et al., “Adherence to Treatment in Allergic Rhinitis During the Pollen Season in Europe: A MASK‐Air Study,” Clinical and Experimental Allergy 55, no. 3 (2025): 226–238, 10.1111/cea.70004. PMC11908838 39956639

[3] J. Bousquet , R. Murray , D. Price , et al., “The Allergic Allergist Behaves Like a Patient,” Annals of Allergy, Asthma & Immunology 121, no. 6 (2018): 741–742, 10.1016/j.anai.2018.07.034. 30056150

[4] M. Wachs , D. Proud , L. M. Lichenstein , A. Kagey‐Sobokta , P. S. Norman , and R. M. Naclerio , “Observations on the Pathology of the Nasal Priming,” Journal of Allergy and Clinical Immunology 84, no. 4 (1989): 492–501. 10.1016/0091-6749(89)90362-x 2477429

[5] G. Ciprandi , S. Buscaglia , G. Pesce , et al., “Minimal Persistent Inflammation Is Present at Mucosal Level in Patients With Asymptomatic Rhinitis and Mite Allergy,” Journal of Allergy and Clinical Immunology 96, no. 6 Pt 1 (1995): 971–979. 10.1016/s0091-6749(95)70235-0 8543756

[6] G. W. Canonica and E. Compalati , “Minimal Persistent Inflammation in Allergic Rhinitis: Implications for Current Treatment Strategies,” Clinical and Experimental Immunology 158, no. 3 (2009): 260–271, 10.1111/j.1365-2249.2009.04017.x. PMC2792821 19765020

